# Exploring the neurocognitive basis of episodic recollection in autism

**DOI:** 10.3758/s13423-018-1504-z

**Published:** 2018-07-09

**Authors:** Rose A. Cooper, Jon S. Simons

**Affiliations:** 1 Department of Psychology, Boston College, Chestnut Hill, MA USA; 2 Department of Psychology, University of Cambridge, Cambridge, UK

**Keywords:** Autism, Long-term memory, Episodic memory, Recollection, Functional connectivity

## Abstract

Increasing evidence indicates that the subjective experience of recollection is diminished in autism spectrum disorder (ASD) compared to neurotypical individuals. The neurocognitive basis of this difference in how past events are re-experienced has been debated and various theoretical accounts have been proposed to date. Although each existing theory may capture particular features of memory in ASD, recent research questions whether any of these explanations are alone sufficient or indeed fully supported. This review first briefly considers the cognitive neuroscience of how episodic recollection operates in the neurotypical population, informing predictions about the encoding and retrieval mechanisms that might function atypically in ASD. We then review existing research on recollection in ASD, which has often not distinguished between different theoretical explanations. Recent evidence suggests a distinct difficulty engaging recollective *retrieval* processes, specifically the ability to consciously reconstruct and monitor a past experience, which is likely underpinned by altered functional interactions between neurocognitive systems rather than brain region-specific or process-specific dysfunction. This integrative approach serves to highlight how memory research in ASD may enhance our understanding of memory processes and networks in the typical brain. We make suggestions for future research that are important for further specifying the neurocognitive basis of episodic recollection in ASD and linking such difficulties to social developmental and educational outcomes.

## Introduction

Memory is an integral part of our daily lives, from indirect influences on our thoughts and behavior to conscious recall of previous experiences, and is a central aspect of development, learning, and social communication. Understanding how and why memory may differ in various populations, including individuals with healthy aging, patients with brain lesions, and children and adults with developmental disorders such as autism spectrum disorder (ASD), is of vital importance for optimizing health and wellbeing in these individuals, and can often provide novel insights into how memory operates in the typical brain.

People diagnosed with ASD most notably have difficulty with social interaction, including the processing of social-emotional cues as well as verbal and non-verbal communication. Additionally, people with ASD commonly exhibit inflexible behavior, fixated interests, and hypersensitivity to sensory input, as defined by the DSM-5 diagnostic criteria. Therefore, flexibly adapting to new environments and cognitive demands can be extremely challenging for these individuals. In addition to the core characteristics of ASD, early research also identified relatively consistent memory impairments, leading to the initial perception of ASD as a form of amnesic disorder (Boucher & Warrington, 1976).

Our understanding of memory functioning in ASD has now developed substantially, with research exploring the pattern of memory difficulties and strengths in this population due to the potential implications for both social communication and education outcomes. Such research has revealed a pattern of relatively unaffected implicit memory, semantic memory, and recognition memory, particularly in individuals without language or intellectual impairments, but diminished episodic recollection, in terms of the ability to retrieve and re-experience the specific details and spatial-temporal context of a previous event (see Boucher et al., 2012; Bowler et al., 2011 for previous reviews). A handful of theoretical accounts have been proposed that attempt to explain why memory of episodic experiences is diminished in ASD; however, the neurocognitive basis of this ability remains largely unclear. This is partly because much research to date has not systematically tested the nature of recollection in ASD with consideration of the different encoding and retrieval processes underlying recollection in the neurotypical population. Integrating these two avenues of research provides novel insights into the neurocognitive basis of memory differences in ASD and raises questions concerning the previous theoretical accounts of memory functioning in this population. Moreover, studying memory in ASD can provide a unique perspective into the nature of memory itself, potentially revealing fresh insights into the role of specific brain networks in memory in the neurotypical population.

In this review, we provide a brief overview of the cognitive neuroscience of recollection, focusing on important encoding and retrieval processes that are most relevant to understanding memory in ASD. We then evaluate research into recollection in ASD and proposed theoretical explanations, as well as recent research that challenge these views and emphasizes the need for a more systematic, integrative approach. Finally, we consider important future investigations that we believe will advance research in this area.

## Encoding and retrieval processes of recollection

Episodic memory refers to our ability to recall and re-experience specific episodes that have a unique spatial-temporal context and involves “autonoetic awareness,” requiring self-reflection (Tulving, 1985). According to dual-process theories, episodic recollection involves controlled search for and evaluation of contextual details (Yonelinas, 2002) and is a threshold “some-or-none” process, where either some qualitative information about an event is recollected, with varying degree of specificity (Onyper et al., 2010), or no context information is recollected. In contrast, familiarity involves the feeling of knowing that something has been encountered before, but without recollection of additional, specific details from the original experience (Yonelinas, 2002). Familiarity is considered to be an automatic process wherein a stimulus is associated with a processing fluency strength, which can give rise to an explicit familiarity-based judgement if a criterion for recognition is surpassed (e.g., Green & Swets, 1966). Measuring recollection necessarily involves measuring the ability to *retrieve* episodic details, involving the search for, reconstruction, and evaluation of a memory, but successful recollection is also reliant on how effectively information was encoded, involving the perception, integration, and transformation of sensory features into a memory representation. If either of encoding or retrieval is dysfunctional, then episodic recollection can be substantially diminished.

Recollection disproportionately benefits from an elaborative, deeper level of processing, such as greater imageability and relating a stimulus to a meaningful context (e.g., Gardiner et al., 2001; Leshikar & Duarte, 2012; Skinner & Fernandes, 2010; Yonelinas, 2001). These effects are likely related to enhancing attention during encoding, given that dividing attention, by using an unrelated task to limit attention to the encoded stimulus, disproportionately impairs subsequent recollection relative to familiarity (Gardiner et al., 2001; Yonelinas, 2001). These effects on recollection are generally observed to be governed by top-down control processes mediated by lateral prefrontal cortex (Blumenfeld & Ranganath, 2007; Dennis et al., 2015; Otten, 2007; Park & Rugg, 2011). However, encoding involves not only directing attention to and processing information, but also the formation of arbitrary and flexible relations between constituent aspects of an experience (Konkel & Cohen, 2009). This “relational binding” process is widely thought to be facilitated by the hippocampus (Diana et al., 2007; Horner et al., 2015; Konkel & Cohen, 2009; Shimamura, 2010), where hippocampal activity during encoding predicts subsequent recollection (Otten, 2007; Park & Rugg, 2011; Ranganath et al., 2003). In contrast, successful encoding of item-specific features and individual objects can be supported by distinct neocortical regions such as inferior occipital/temporal cortex (Horner et al., 2015) and the perirhinal cortex (Awipi & Davachi, 2008; Davachi, 2006). The hippocampal binding process involves the integration of signals from sensory-specific brain regions into a bound representation (Preston & Eichenbaum, 2013), while frontally-mediated encoding processes are thought to modulate the degree to which elements are processed and integrated within the hippocampus (cf. Addis & McAndrews, 2006).

Even if encoding is optimal, the context in which an event is retrieved can also have a separable influence on the likelihood of recollection success. Retrieval cues narrow the information to be searched for and monitored in memory (Morcom & Rugg, 2012), promoting recollection success by increasing the overlap between encoding and retrieval contexts (Elfman & Yonelinas, 2015), as described by the encoding specificity principle (Tulving & Thomson, 1973). Recollection is a reconstructive process (Schacter & Addis, 2007) that is thought to depend on the ability of the hippocampus to engage in pattern completion and integrate distinct aspects of a memory trace (McClelland et al., 1995; Moscovitch, 2008). As such, greater hippocampal activity during context recollection compared to item familiarity is widely observed (see Eichenbaum et al., 2007; Rugg & Vilberg, 2013 for reviews; see Kim, 2016; Skinner & Fernandes, 2007; Spaniol et al., 2009 for meta-analyses). Recent theories emphasize that the role of the hippocampus is best described by its representational content rather than memory retrieval processes (Cabeza & Moscovitch, 2013; Cowell et al., 2010; Diana et al., 2007), where the hippocampus is sensitive to relational rather than item representations regardless of whether encoding and/or retrieval is explicit or implicit (Dew & Cabeza, 2011; Duss et al., 2014; Hannula & Greene, 2012; Olsen et al., 2012; Reber et al., 2012).

Additional brain regions, such as prefrontal and parietal cortices, are required to facilitate the conscious experience of recollection (Ranganath, 2010; Cabeza & Moscovitch, 2013; Hannula & Ranganath, 2009; Moscovitch, 2008; Moscovitch et al., 2016). The posterior parietal cortex in particular has been proposed to play an important role in explicit memory reactivation (Rugg & Vilberg, 2013), which is suggested by the consistent decreases in recollection confidence and vividness exhibited by patients with parietal lesions (Hower et al., 2014; Simons et al., 2010). It has been theorized that posterior parietal cortex is directly involved in the online representation and integration of event-specific information (Bonnici et al., 2016; Kuhl & Chun, 2014; Vilberg & Rugg, 2012). Moreover, activity of the angular gyrus has been observed to track the perceived vividness and objective precision of retrieved memories (Kuhl & Chun, 2014; Richter, Cooper, et al., 2016). The ability to engage in recollection and reflect upon these retrieved memory representations is dependent upon strategic retrieval processes mediated by lateral prefrontal cortex (Badre &Wagner, 2007; Simons & Spiers, 2003), including pre-retrieval cue specification (Dobbins et al., 2002; Moss et al., 2005) and post-retrieval monitoring (Dobbins et al., 2002; Gallo et al., 2010). The medial prefrontal cortex on the other hand has been associated with introspective processes such as reality monitoring, self-referential processing, metacognition, and contextual integration (Buckner & Carroll, 2007; Preston & Eichenbaum, 2013; Simons et al., 2017). A core function of the medial prefrontal cortex is thought to be mental simulation – shifting from our present perspective to an alternative, internally represented perspective – broadly characterised as “self-projection” (Buckner & Carroll, 2007). The medial prefrontal cortex is considered to integrate retrieved memories with current goals, states, and inter-related memories by highlighting and distinguishing between “meaningful” contexts (Schlichting & Preston, 2015; Zeithamova et al., 2012). Therefore, hippocampal relational processes facilitate reinstatement of episodic memories (Gordon et al., 2014; Ritchey et al., 2013), but are crucially accompanied by distinct parietal and frontal processes that involve consciously representing a past experience and integrating it with prior knowledge.

It is important to emphasize, however, that episodic recollection is determined by the flexible coordination of these different cognitive processes and neural systems (Cabeza & Moscovitch, 2013). Functional connectivity strength, over and above region-specific activity, of whole-brain networks involving important hubs such as the hippocampus and medial prefrontal cortex, is important for episodic recollection (Geib et al., 2015; King et al., 2015; Schedlbauer et al., 2014; Robin et al., 2015; see Preston & Eichenbaum, 2013 for a review), reflecting increased transfer and integration of information that promotes recollection success. Hippocampal connectivity with regions of the fronto-parietal control network (FPCN) is enhanced during successful and vivid recollection (Bowman & Dennis, 2016; Ford & Kensinger, 2016; Hannula & Ranganath, 2009; Wais et al., 2010). Increases in connectivity between the default mode network (DMN) and FPCN also facilitate flexible goal-directed behavior (Spreng et al., 2010) that contributes to episodic memory retrieval (Fornito et al., 2012). These studies highlight the importance of functional interactions over and above distinct neurocognitive processes to our understanding of episodic recollection.

## Episodic recollection in ASD

While it has become clear that people with ASD are generally far from amnesic, and can often have good memory for particular types of information, individuals with ASD, without accompanying language or learning difficulties, tend to exhibit a characteristic pattern of memory performance. Specifically, episodic recollection appears to be disproportionately impaired (see Bowler et al., 2011; Boucher et al., 2012) over and above semantic memory, familiarity-based recognition memory, and implicit memory, which often show minimal differences between people with ASD and neurotypical controls (e.g., Bowler et al., 1997; Gardiner et al., 2003; Hedley et al., 2012). For instance, direct comparisons of episodic and semantic memory reveal clear reductions in the former in the presence of good ability for the latter: Gaigg et al. (2014) observed that ASD participants have good semantic knowledge for the chronological order of historical figures but have difficulty recalling the experiment-specific temporal order in which the same historical figures had been studied. Similarly, Crane and Goddard (2008) demonstrated that adults with ASD possess the same level of explicit semantic self-knowledge as do typical adults but have selectively reduced autobiographical episodic memory recall. The following summary highlights the types of experiments that have been used to test episodic recollection in ASD, which demonstrate a relatively consistent difficulty in retrieving specific details of past events.

Several studies have assessed personal autobiographic recollection in ASD, often quantifying specific and general memory details. In these studies, ASD participants exhibit a reliable reduction in explicit recall of event-specific autobiographic episodic memory details (e.g., Bruck et al., 2007; Crane et al., 2012; Goddard et al., 2007; Lind & Bowler, 2010; Tanweer et al., 2010), but they can often recall just as many general event details as typical individuals (Crane et al., 2009; Maister et al., 2013), suggesting an increase in the extent to which personal memories are “factual”. Such studies have been complemented by experimental paradigms where participants are tested on their ability to recollect the context of studied stimuli. Subjective remember/know judgements are thought to map well on to recollection and familiarity processes in recognition memory tasks (Yonelinas, 2002), with the former based on memory for details of the context in which the item was studied, and the latter reflecting item familiarity but an inability to recall details of the original encoding experience (Tulving, 1985). Subjects with ASD are consistently less likely than typical participants to report that they “remember” contextual details of an item but are just as likely or more likely to report that they “know” they studied an item before (Bowler et al., 2000; Bowler et al., 2007; Cooper et al., 2015; Cooper et al., 2017a; Gaigg et al., 2015; Meyer et al., 2014; Souchay et al., 2013; Tanweer et al., 2010). However, some studies have also observed reduced familiarity-based recognition memory in ASD (Bowler et al., 2004; Solomon et al., 2016), a finding that is more commonly identified in individuals with language or learning impairments (Boucher et al., 2008; Bigham et al., 2010). Nonetheless, remember/know reports provide convincing evidence that the overall subjective experience of recollection occurs less frequently in individuals with ASD compared to neurotypical individuals.

In support of the findings from subjective methods, source memory tasks in ASD have provided additional evidence for a reduction in episodic recollection. These tasks assess participants’ recollection objectively by manipulating the context or “source” in which stimuli are studied (Johnson et al., 1993). People with ASD have often been reported to exhibit difficulties with source memory, including temporal order (Bennetto et al., 1996; Gaigg et al., 2014), location (Bowler et al., 2004; Cooper et al., 2015; Ring et al., 2015), as well as memory for whether information was internally or externally generated at encoding (Cooper et al., 2016; Hala et al., 2005; Lind & Bowler, 2009; Maras et al., 2013; Russell & Jarrold, 1999), but participants with ASD generally exhibit typical item recognition memory in these studies. Similarly, people with ASD demonstrate reduced associative memory for object-feature associations (Bowler et al., 2014; Massand & Bowler, 2012) and word-object pairs (Southwick et al., 2011). However, impaired source memory in individuals with ASD has not always been observed. For example, Souchay et al. (2013) identified no difference between adolescents with ASD and neurotypical controls when asked to recall the color, location, and temporal order of studied information. Similarly, Bowler et al. (2015) observed no overall difference between their groups for spatial and temporal source memory, and some studies have identified no differences in the ability of individuals with ASD to recall whether they or someone else performed an action (Farrant et al., 1998; Grainger et al., 2014a; Hill & Russell, 2002; Zalla et al., 2010). Amongst these latter studies, the number of participants and number of trials per condition has often been very small, emphasizing that such underpowered studies are unlikely to detect the relatively subtle source memory deficits in ASD (cf. Cooper et al., 2016; Lind & Bowler, 2009). However, contradictory evidence across long-term memory tasks in ASD may also serve to highlight the possible heterogeneity of memory performance in people with ASD.

Variability in performance on tasks measuring episodic recollection in ASD has also been proposed to be a function of “task support” (Bowler et al., 2004), where explicit recollection difficulties in ASD are observed and accentuated with low retrieval support, such as minimal retrieval cues, and when information to be recalled requires a high level of organization. Minimal retrieval cues and high organization demands are often characteristic of tasks assessing subjective recollection over and above experiments testing memory for single contextual details cued in source memory tasks. In support of this hypothesis, spatial source memory in ASD is disproportionately improved following retrieval support, where visual source cues were provided to participants (Bowler et al., 2004; Bowler et al., 2015). Interestingly when provided with retrieval cues, such as a picture of the room in which a crime was committed (Maras & Bowler, 2012) or with questions targeting specific details of autobiographical events (Crane et al., 2013), individuals with ASD can recall episodic information with the same level of specificity as neurotypical individuals. Maras and Bowler (2012) thus claimed that people with ASD have difficulty mentally reinstating an event context but can often exhibit accurate memory for the event details when provided with physical cues. Memory retrieval in ASD seems likely, therefore, to be overly context dependent (cf. Tulving & Thomson, 1973) compared to memory retrieval in typical individuals, with an interesting case study by Boucher (2007, pp. 256-257) reflecting this recollection difference via the experience of a person with ASD (JS):

*“*JS describes his capacity for voluntary retrieval as state dependent. ... JS visits the UK quite regularly, arriving at Heathrow. However, when he sets out on his journey, or whilst on the aeroplane, he cannot recall Heathrow, or any details of how to travel from Heathrow to his destination in the UK (he does, of course, have instructions written down). He has no memory of previous visits, of where to find the shuttle train service to London, or the bus to the hotel, until he has arrived at Heathrow and recognizes something which then cues a memory of previous visits. Thus, both recognition and cued recall are superior to free recall. Of course, some degree of state dependency is a common factor in retrieval for everyone. However, whereas for most people dependency is far from total, for JS it can be close to total. To remember events ..., such as the activities of a trip abroad, JS formulates an account verbally reconstructed from a series of facts such as ‘Arrived at Paris on Friday evening; took a taxi to the hotel; met X in the bar; had a meal; went to bed.’ The order of these activities is also reconstructed by analytic reasoning.”

While JS’s memory experience may be towards the more extreme end of recollection observed in individuals with ASD, it provides an interesting insight into how this dysfunction might manifest in everyday situations. The current perspective on episodic recollection in ASD is that altered recollective experience arises from differences in the way in which memory representations are manipulated, rather than deficits in memory for the information itself (Bowler et al., 2011). Thus, details of events may well be encoded to some extent in ASD but the *way* in which they are encoded, stored, and reconstructed could lead to difficulty recollecting information with the same level of detail as seen in neurotypical individuals. A collection of theories has been proposed that attempt to explain the basis of episodic recollection differences in ASD, each of which focuses on a different subset of neurocognitive processes. Though these theories may account for particular features of memory in ASD, no theoretical approach alone provides a full explanation of the episodic recollection differences observed in this population. Notably, the accounts were not proposed to be mutually exclusive or all-encompassing, but, equally, different theories of memory dysfunction in ASD often have not been distinguished from one another experimentally. Below, we consider the evidence supporting prominent conceptualizations of episodic recollection in ASD to date, as well as the potential limitations of evidence underlying each account. Moreover, we emphasize the importance of distinguishing between distinct encoding and retrieval processes and how studying *interactions* between different neural systems will likely clarify the basis of episodic recollection in ASD.

## Neurocognitive accounts of recollection in ASD

### Self-projection

One approach to explaining recollection in ASD focuses on the intrinsically self-oriented nature of the experience, specifically the requirements of autonoetic awareness (Tulving, 1985), and the ability of people with ASD to be aware of and reflect upon past, present, or future perspectives of themselves and other people (Lind & Bowler, 2010). Lind (2010) proposed that diminished perspective taking and self-awareness in ASD (Lombardo & Baron-Cohen, 2011; Williams, 2010), often associated with atypical or attenuated medial prefrontal neural activity in these individuals (Kennedy & Courchesne, 2008; Lombardo et al., 2009), underpin the reduced ability to encode, and subsequently recollect, self-related and social information. In support of this proposal, people with ASD have been observed to exhibit a reduced self-reference effect in memory (Grisdale et al., 2014; Henderson et al., 2009; Lombardo et al., 2007), in which neurotypical individuals exhibit enhanced memory for stimuli when encoded in relation to the self compared to focusing on perceptual characteristics, for example. Conversely, other studies have indicated an intact benefit of self-related encoding in ASD (Cooper et al., 2016; Grainger et al., 2014a; Lind & Bowler, 2009; Williams & Happe, 2009). These contrasting results have been suggested to reflect typical physical self-awareness in ASD alongside atypical psychological self-awareness (Lind, 2010; Williams, 2010). Moreover, some evidence has suggested that autobiographical episodic memories in ASD are less organized around self-goals (Crane et al., 2009) and are less likely to be retrieved from a first-person perspective (Lind & Bowler, 2010; Lind et al., 2014). Interestingly, individuals with ASD also show similar difficulties with episodic future thinking (Lind & Bowler, 2010; Lind et al., 2014), which could reflect the common requirement of self-projection (cf. Benoit & Schacter, 2015; Buckner & Carroll, 2007).

Individuals with ASD also show signs of reduced integration of social information into their episodic memories, exhibiting greater impairments in recollection of social details relative to other perceptual contextual details (O’Shea et al., 2005) as well as disproportionately reduced memory for socially-encoded words compared to perceptually-encoded words (Brezis et al., 2014; Henderson et al., 2009; Lombardo et al., 2007). Researchers have therefore suggested that a weakness in processing or prioritizing self and social information mediated by medial prefrontal cortex function, rather than a separable memory deficit per se, leads to subsequent difficulties in the recollection of specific events details in ASD (Ben Shalom, 2009; Brezis, 2015). While it is highly probable that individuals with ASD experience some difficulties in processing self- and socially-related information, the current evidence to date does not provide definitive support for this explanation of recollection difficulties (cf. Brezis et al., 2014).

For instance, two source memory studies have observed that participants with ASD are equally impaired in their recollection of perceptual contextual details as their recollection of self and social information (Cooper et al., 2016; Hala et al., 2005). Furthermore, the proposal of difficulties with self-projection is primarily based on studies of autobiographic episodic recollection (e.g., Crane & Goddard, 2008; Lind & Bowler, 2010), placing an emphasis on recollecting self-oriented events, but recent evidence pointing to difficulties in recollecting basic visual associations and visual details of experimental stimuli (e.g., Bowler et al., 2014; Cooper et al., 2015) questions the extent to which self-projection and social-information processing could account for attenuated recollection in ASD. A final important point is that self and social information could simply be more difficult or “complex” to encode and retrieve, requiring higher level representations relative to semantic and perceptual information, for example. This possibility is rarely considered or controlled for. Therefore, it is unknown whether a reduced ability to engage in self-projection and process self-related and social information, mediated by medial prefrontal dysfunction, can explain recollection differences in ASD or whether difficulties with memory organization and elaborative processing more generally might alternatively explain the aforementioned findings.

### Complex information processing

An alternative attempt to characterize memory functioning in ASD has focused on impairments in “complex information processing” (Minshew & Goldstein, 2001), highlighting that memory performance in ASD can be just as high as in neurotypical individuals but disproportionately decreases as the conceptual structure of the material to be learnt and the retrieval task increase demands on cognitive control (Minshew & Goldstein, 2001; Williams et al., 2006; Williams et al., 2017). This pattern is clearly evident in ASD, with memory generally functioning well when information can be implicitly retrieved or recognized based on familiarity but suffering when recollection is required, particularly in cases of minimal retrieval cues and a more “complex” organization of material to be remembered, such as autobiographical recall. Minshew and Williams (2007) frame this account in terms of aberrant frontal neural connectivity, which they claim places limits on the coordination of neural systems and the degree to which information can be integrated. Altered prefrontal cortex connectivity has been argued to contribute to several characteristics of ASD (Courchesne & Pierce, 2005; Just et al., 2012), and a number of studies have demonstrated reduced functional connectivity in ASD between frontal and parietal regions involved in top-down control (Damarla et al., 2010; Solomon et al., 2009) and attenuated lateral prefrontal activity during executive function and perceptual tasks (Damarla et al., 2010; Koshino et al., 2008; Solomon et al., 2015). Moreover, a complex information processing account parallels the general executive function approach (e.g., Hill, 2004), which has been argued by a number of researchers to provide a good explanation of attenuated episodic recollection observed in this population (e.g., Hala et al., 2005; Solomon et al., 2016).

Specifically, some behavioral studies have identified a stronger relationship between executive function and recollection in individuals with ASD compared to typical controls (Goddard et al., 2014; Maister et al., 2013), perhaps suggesting a greater interdependence between these cognitive processes in ASD, though Semino et al. (2017) recently observed that basic executive function measures could not account for source memory performance in adults with ASD. Conversely, Solomon et al. (2016) observed that cognitive control demands, rather than relational processing demands, appear to have the greatest influence on memory performance in adolescents with ASD. Relatedly, there is also evidence that recollection in ASD may not benefit from deliberate, strategic encoding (Meyer et al., 2014), and the benefit of task support, such as the way that retrieval cues improve recall and source memory in ASD (Bowler et al., 2004; Maras et al., 2013), could further highlight the influence of cognitive control demands on memory performance. In particular, Williams et al. (2017) observed that adults with ASD had difficulty using organizational strategies to facilitate episodic recollection. However, some evidence suggests that recollection in ASD cannot be fully explained by reduced ability to engage strategic encoding processes, as individuals with ASD have been found to exhibit typical enhancements of intentional over incidental encoding on subsequent recollection in the presence of an overall reduction in recollection frequency (Cooper et al., 2017a; Souchay et al., 2013).

The limited number of fMRI studies investigating long-term memory in ASD thus far have hinted at atypical lateral frontal function during memory encoding, perhaps reflecting altered organization of material to be learnt (Gaigg et al., 2015; Greimel et al., 2012), and during memory retrieval (Cooper et al., 2017b), possibly reflecting a difficulty engaging top-down strategic retrieval processes. Additionally, two EEG studies have observed attenuated frontal ERPs across all time points during memory retrieval in ASD with old/new effects being more posteriorly located than in typical individuals (Massand & Bowler, 2012; Massand et al., 2013), which may reflect dysfunctional strategic retrieval processes. Hence, it is possible that atypical frontal functioning and frontal-posterior integration mediating top-down “complex information processing” may contribute to altered recollection in ASD.

However, this account remains too underspecified, both in terms of theoretical explanation and the diversity of experiments used to support it (cf. Bowler et al., 2011). First, a general complex information processing theory does not provide sufficient explanation of exactly which strategic or organizational memory processes people with ASD find most challenging, and whether differences are most apparent during memory encoding and/or memory retrieval. Secondly, it is important to highlight that previous studies have varied widely in the types of tasks and instructions used to promote strategic or organizational memory processes and measure executive function or cognitive control, and have often not defined exactly what “strategic” mechanisms might be most compromised in ASD. Such inconsistencies are likely to contribute to the mixed findings to date. It is thus vital for future research to test *specific* encoding and retrieval processes to pinpoint the basis of attenuated episodic recollection in “complex” tasks in ASD. A further tentative argument against this broad account that has been put forward is that memory difficulties in ASD do not mimic those of frontal lobe patients (Bowler et al., 2010), although of course this by no means rules out the possibility that some frontal-related processes influence the nature of long-term memory in ASD. It does, however, raise the likely possibility that dysfunction of other neurocognitive systems needs to be taken into account.

### Relational binding

A reduction in hippocampal relational binding was proposed as a potential clarification of the complex information processing hypothesis. According to this view, memory impairments in ASD arise from a reduced tendency to utilize relations between items to “bind” features of an event together in memory, leading to a reduction in memory for the specific “relational” contextual information that forms the basis of episodic memory, but typical or even superior (compensatory) item-specific memory processes (Gaigg et al., 2008; Bowler et al., 2011; Bowler et al., 2014). One form of evidence that has often been used to support the relational binding account comes from conceptual organization of words during recall, which some evidence suggests is related to hippocampal activity during encoding in neurotypical adults (Addis & McAndrews, 2006). Specifically, people with ASD can demonstrate just as good an ability to recall lists of unrelated items but show reduced recall of conceptually related stimuli compared to neurotypical controls (Bowler et al., 1997; Bowler et al., 2008; Gaigg et al., 2008; Maister et al., 2013). There is also evidence for deficits in organization of episodic memory recall around self-related and semantic contexts in ASD (Crane et al., 2009; Loth et al., 2011). This research could suggest that recollection differences in ASD may be associated with poor memory organization (Bowler et al., 2008) and thus may be more readily apparent when a relational framework is required or beneficial for successful memory performance.

Problematically, however, difficulty recalling conceptually organized material has not always been demonstrated in ASD (Beversdorf et al., 1998; Bowler et al., 2009; Mottron et al., 2001; Whitehouse et al., 2007), and some studies have interestingly found that reductions in memory retrieval are not moderated by conceptual organization of material to be learnt (Bowler et al., 2009; Carmo et al., 2016; Gaigg et al., 2015; Smith et al., 2007). Moreover, any difficulty observed in recall of related information also appears to be reduced in tasks that place fewer demands on recollection processes: individuals with ASD can show a recognition advantage for conceptually related words, as do typical controls (Bowler et al., 2008; Toichi et al., 2002), and can show typically enhanced cued recall following semantic than perceptual encoding of words (Gardiner et al., 2003; though see Toichi & Kamio, 2002). Additionally, individuals with ASD may be just as susceptible to conceptual false memories, in terms of falsely remembering a word based on its semantic similarity to studied words (Bowler et al., 2000; Gardiner et al., 2003; Kamio & Toichi, 2007; though see Beversdorf et al., 2000), and can also show schema-consistent misinformation effects during event recall (Bruck et al., 2007; Maras & Bowler, 2011). Therefore, it has been argued that people with ASD are perhaps aware of, and encode, the relational structure of information to be learnt, reflecting an “intact” semantic encoding system (Carmo et al., 2016), but can have difficulty using such an organizational structure to freely reconstruct a past event.

Howewer, the true basis of these relational processing effects cannot be readily determined given that semantic encoding and organization has been more commonly associated with lateral prefrontal activity and not hippocampal binding processes in the neurotypical population (e.g., Demb et al., 1995; Otten et al., 2001; Simons & Spiers, 2003). This point serves to highlight that relational processing, involving the use of semantic organizational strategies (Hunt & Seta, 1984), has often been conflated with the idea of hippocampal relational binding, which is the ability to bind constituent aspects of an experience together into a flexible, unique representation (Konkel & Cohen, 2009), in the ASD literature to date. Interestingly, a behavioral study that aimed to directly test the relational binding hypothesis assessed memory for arbitrary item-context conjunctions, and for the item or context elements alone, and observed that the ASD group exhibited typical levels of recognition of single item or context elements but reduced recognition of item-context conjunctions (Bowler et al., 2014). However, tasks such as this can be confounded by other differences between relational and item memory task conditions, such as difficulty and demands on recollection and familiarity (e.g., Bowler et al., 2014), recall versus recognition test procedures (e.g., Bowler et al., 2004; Massand et al., 2013), and the specificity of memory details or the amount of information that needs to be encoded and retrieved (e.g., Bowler et al., 2014; Maister et al., 2013).

Furthermore, recent studies testing the relational binding hypothesis using more comparable item and relational memory tests have not provided definitive support for a disproportionate binding difficulty in ASD. One study (Cooper et al., 2015) adapted a task that equates the difficulty and specificity of item and relational memory (Hannula et al., 2010) to include remember/know judgements and observed an equal reduction in recollective retrieval of both item-specific features and relational spatial information, but not in familiarity-based memory. A disproportionate impairment in relational memory would be expected following a hippocampal binding deficit (cf. Cowell et al., 2010) and is observed on this task in patients with hippocampal lesions (Hannula et al., 2015). Relatedly, Ring et al. (2016) used a task developed by Konkel et al. (2008) that also revealed disproportionate relational binding impairments in hippocampal lesion patients, and similarly observed comparable item and relational memory impairments in adults with ASD (also see Solomon et al., 2016). However, it is important to stress that neither of the aforementioned studies manipulated or controlled for encoding strategies, and it is thus possible that participants with ASD did not spontaneously engage in elaborative, relational encoding of both item-specific and relational stimuli compared to control participants.

Neuroimaging studies of memory in ASD have also provided limited evidence to date of specific hippocampal dysfunction. Interestingly, a recent study that investigated the link between memory and hippocampal structure in ASD found no evidence of differences in hippocampal volume between individuals with ASD and neurotypical controls and no correlation between hippocampal volume and memory function (Trontel et al., 2015). Moreover, fMRI studies to date have observed minimal evidence for differences in hippocampal activity during memory encoding in ASD (Cooper et al., 2017b; Gaigg et al., 2015; Solomon et al., 2015), thus questioning the link between altered hippocampal relational encoding mechanisms and subsequent recollection in this population. However, Bowler et al. (2011) have emphasized that any relational binding difficulties might not emerge directly from hippocampal dysfunction per se, but rather altered connectivity between the hippocampus and cortical regions.

Of course, if relational binding difficulties could underlie recollection impairments in ASD, then reduced binding should also be apparent on tasks that are not restricted to recollective memory retrieval. Beyond memory, other abilities have also been linked with hippocampal relational binding processes including episodic future thinking, fictitious scene construction, and spatial navigation (Eichenbaum et al., 2016; Mullally & Maguire, 2014) and, in potential support of the relational binding perspective, all appear to be impaired in individuals with ASD (Lind & Bowler, 2010; Lind et al., 2014; Lind et al., 2013). Moreover, a recent study by Ring et al. (2017b) observed that individuals with ASD exhibited reduced performance only in structural learning of spatial information but not other types of visual discrimination, possibly tapping into hippocampal function (cf. Aggleton et al., 2007), which could not be accounted for by measures of executive ability. Of note though, without congruent neural evidence, the basis of this difference in task performance cannot be assumed (the reasons for which are discussed at the end of this section). Interestingly, however, any relational memory impairments may not extend beyond explicit memory in ASD; even when individuals with ASD exhibit impaired explicit relational memory – identifying the location previously associated with an object – the same participants were just as good as controls at implicit relational memory (Ring et al., 2015), thus indicating a separable difficulty engaging in explicit recollection and perhaps not in basic relational binding. Therefore, while altered relational processing mechanisms potentially contribute to differences in recollective experience in ASD, relational binding deficits may not be able to account for the reduced ability to reconstruct and re-experience past events as observed in this population.

### Subjective experience of recollection

It has been suggested that the hippocampus plays a role in binding but not in the experience of explicit recollection during retrieval, which is linked to posterior parietal cortex (Moscovitch et al., 2016). Some researchers have focused on long-term memory in ASD as reflecting differences in the subjective, conscious experience of recollection specifically (Bigham et al., 2010; Boucher et al., 2008). In particular, these authors have suggested that posterior parietal dysfunction might provide a good explanation of recollection impairments in ASD (Boucher & Mayes, 2012), largely based on comparisons to memory performance exhibited by parietal lobe patients. For instance, evidence clearly suggests that individuals with ASD can exhibit pronounced reductions in subjective recollection measures, in particular, such as specific autobiographical recall (e.g., Crane et al., 2012; Lind & Bowler, 2010), remember judgements (e.g., Bowler et al., 2007; Cooper et al., 2015; Meyer et al., 2014), memory confidence (Grainger et al., 2014b), and self-perceived episodic memory salience and quality (Lind & Bowler, 2010; Lind et al., 2014), as is typically seen following parietal lobe lesions (e.g., Davidson et al., 2008; Drowos et al., 2010; Simons et al., 2010; Yazar et al., 2014).

Moreover, there is evidence that individuals with ASD show impaired metamemory – subjective judgements of mnemonic accuracy – during both recognition memory tasks (Grainger et al., 2014b; Wilkinson et al., 2010; Wojcik et al., 2013) and source memory tasks (Cooper et al., 2016), as has recently been observed in patients with parietal lesions (Ciaramelli et al., 2017). Such differences in metamemory, and the effect on recollective experience, would be difficult for any account predominantly focusing on encoding processes, to explain: for example, a relational binding account would propose that the active process of encoding and subsequently retrieving relationships between elements of an experience should be impaired, but there would be no reason to predict that individuals with ASD should not be able to accurately evaluate the quality of that memory. A metamemory deficit rather emphasizes that individuals with ASD instead, or further, have difficulty reflecting upon a retrieved memory representation, potentially revealing distinct impairments in retrieval mechanisms that contribute to altered recollective experience in this population. However, it is important to note that prefrontal dysfunction, encompassing both self-projection and complex information processing difficulties, could also contribute to suppressed metamemory ability in ASD.

Studies have not consistently observed a difference in metamemory “judgements of learning” in ASD (Grainger et al., 2016; Wojcik et al., 2014), thus questioning the full extent of reduced subjective awareness and its relation to recollection in this population. Although it is possible that parietal dysfunction plays some role in recollective experience in ASD, a clear area of divergence between parietal patients and people with ASD is that the former tend not to show any changes in objective source memory (e.g., Simons et al., 2010). A recent fMRI study of recollection in ASD also found no evidence of possible parietal dysfunction on both behavioral and neural levels (Cooper et al., 2017b). However, even though parietal dysfunction *alone* may be unlikely to fully explain recollection in ASD, the possible contribution of parietal processes to recollection dysfunction remains unclear and largely untested and more research is needed to investigate this possible connection (Boucher & Mayes, 2012).

### Summary

Each of the accounts discussed here may explain certain aspects of memory functioning in ASD, but none alone is likely to explain the full extent of differences in episodic recollection in this population. More importantly, however, the majority of existing studies of recollection in ASD are small and underpowered, and have often not taken a systematic approach of targeting specific encoding and retrieval mechanisms that might drive recollection dysfunction. Thus, experimental findings can often be explained by multiple theoretical perspectives, which does not allow claims to be made about an underlying mechanism. Furthermore, each of the proposals discussed thus far has leaned towards a particular brain region or single neurocognitive mechanism as an explanation of memory dysfunction in ASD, even though most theoretical accounts have also acknowledged that episodic recollection in ASD will likely be best explained by adopting an integrative approach, considering functional interactions between different brain regions and cognitive processes. This is particularly likely in the case of recollection (and any complex cognitive process) given the broad network of regions involved in coordinating both encoding and retrieval (cf. Rugg & Vilberg, 2013).

Going beyond recollection, the general neuroscientific approach to ASD in recent years has placed much more emphasis on integration and widespread differences in functional connectivity than region-specific dysfunction (Just et al., 2012), most notably in tasks relying on higher level cognitive processes (Kana et al., 2011). Specifically, long-range underconnectivity is found particularly during cognitively demanding tasks in ASD, with differences also apparent in task-specific modulations of whole-brain network connectivity (Barttfeld et al., 2012; Uddin et al., 2015). Additionally, ASD is a neurodevelopmental disorder, meaning that the brain has developed differently than in typical individuals, including some neural properties that may be dysfunctional and others that could be compensatory. Therefore, it is by no means straightforward to attribute differences in behavioral tasks between ASD and neurotypical individuals to specific neural mechanisms, because it cannot be assumed that the underlying cognitive and neural processes being utilized are necessarily the same for a given memory task (Mottron et al., 2008). For instance, in an EEG study, Massand et al. (2013) observed no reduction in word recognition memory in ASD, consistent with good familiarity-based retrieval, but reported differences between ASD and typical controls in the magnitude and location of the typical familiarity-related early frontal ERP effect. To begin to address some of these limitations, we consider two recent studies that, using eye-tracking and fMRI, shed further light on the encoding and/or retrieval basis and nature of episodic recollection dysfunction in ASD and provide evidence for a separable difficulty engaging in recollective retrieval that might be best characterized by atypical hippocampal *connectivity* rather than region-specific dysfunction.

## Recollective retrieval and neural connectivity in ASD

Both encoding and retrieval processes are important for successful recollection, with impairments potentially emerging from dysfunction at either stage, or indeed both. It is impossible to determine the neurocognitive basis of recollection in ASD without also attempting to distinguish these two stages of long-term memory. However, establishing whether an item has been encoded can often only be achieved by testing memory for that item later on, meaning that encoding and retrieval processes are difficult to tease apart.

**Fig. 1:**
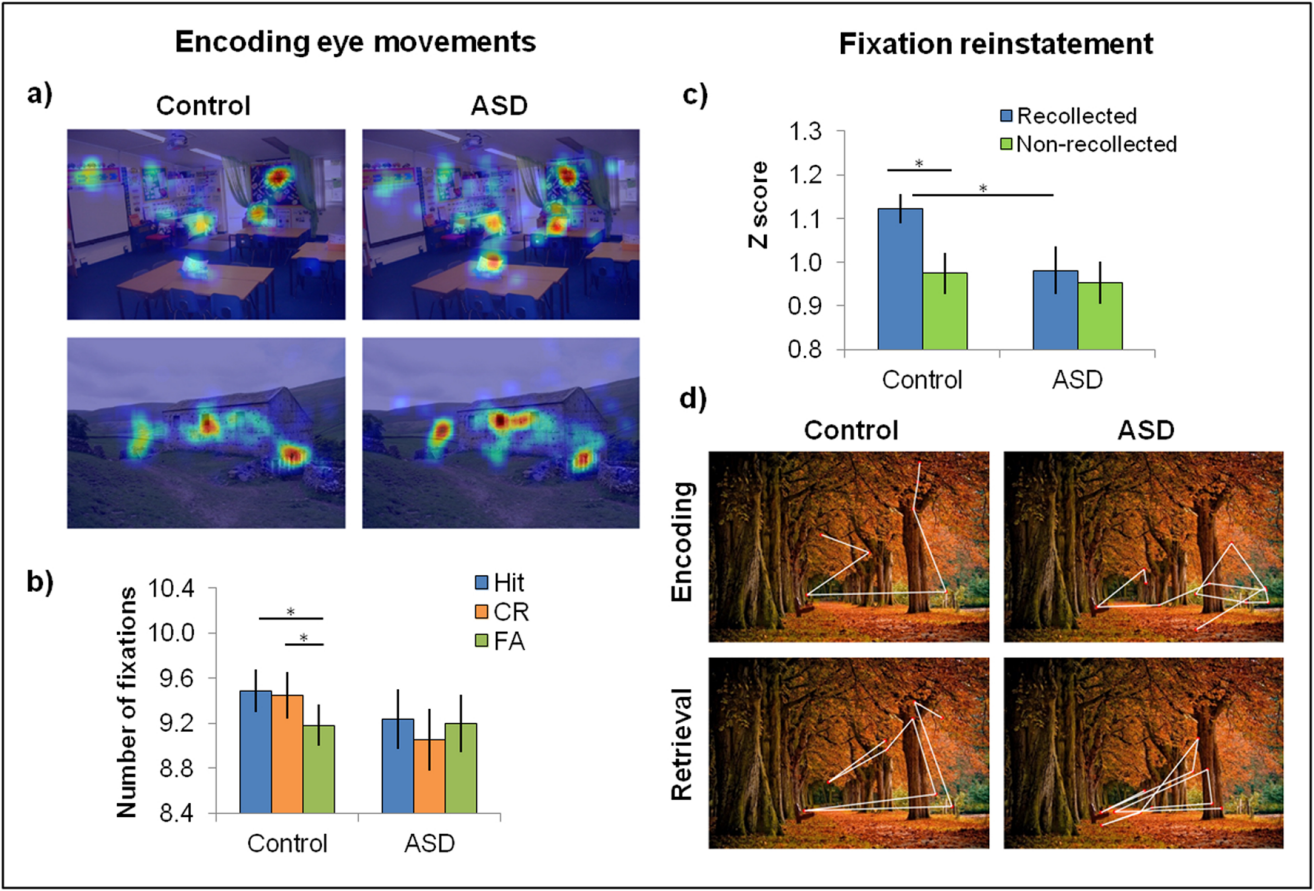
Figure adapted from Cooper et al. (2017a). (**a**) Fixations made to two scenes studied during the memory encoding phase, illustrating the similarity in fixation patterns between the control and ASD groups. (**b**) While the number of encoding fixations did not differ between the groups, encoding fixations only predicted subsequent memory success (Hits and Correct Rejections) relative to false recognition (FAs) in the control group, and not in the ASD group. (**c**) Even when participants reported that they recollected a scene during retrieval, individuals with ASD did not reinstate encoding eye movements to the same degree as control participants did, which is illustrated in part (**d**)

Eye movements can provide valuable information that makes them a particularly useful tool to investigate and distinguish encoding and retrieval processes. In one recent study, adults with ASD and neurotypical control participants studied a series of scene photographs and were subsequently asked to distinguish studied target scenes from similar lure scenes and provide remember/know judgements (Cooper et al., 2017a). The distribution and number of fixations during encoding predicted subsequent recollection in the control group, supporting evidence that fixations reflect the accumulation of evidence and formation of a more detailed representation (Pertzov et al., 2009; Molitor et al., 2014; Kafkas & Montaldi, 2011; Liu et al., 2017). Interestingly, the pattern and number of encoding fixations did not differentiate the ASD and control participants, and an increase in encoding fixations was accompanied by a comparable enhancement of recollection performance following elaborative encoding instructions in the ASD and control groups, indicating similar encoding mechanisms and function of eye movements during encoding. In contrast to the neurotypical adults, however, eye movements during encoding did not predict trial-by-trial subsequent recollection in the ASD group, which was significantly impaired (as also recently observed by Ring et al., 2017a). These findings represent a direct dissociation between observed encoding processes and recollection success in adults with ASD, in which recollection failures were present even for items that were apparently “successfully” encoded (see Fig. 1).

The ASD group were also less likely to reinstate the pattern of encoding eye-movements during recollection than the control group (Cooper et al., 2017a), suggesting a difficulty reconstructing encoded visuo-spatial memory representations (cf. Laeng et al., 2014; Olsen et al., 2014) during retrieval even when successful recollection is reported. These differences emerged despite the finding that the eye movements of individuals with ASD during memory errors indicated correct implicit memory (also see Hedley et al., 2012; Ring et al., 2015), as also observed in neurotypical controls, possibly indicating that the memory representations themselves were present, in some form, but could not be explicitly recollected as successfully in people with ASD.

It is of course possible, though, that there were differences in the neural mechanisms operating at encoding between the ASD and neurotypical groups that were not captured via eye movements by Cooper et al. (2017a). Additionally, with regard to the nature of any deficit in recollective retrieval, previous studies have been unable to determine exactly how memories are retrieved differently in ASD. For example, it is possible that mnemonic information cannot be retrieved with the same level of specificity and precision, which could lead to impaired source memory if the information retrieved is not of sufficient quality to be diagnostic, and might also account for subjective recollection reductions in judgements of salience and confidence (e.g., Grainger et al., 2014b; Lind et al., 2014) in ASD. In contrast, it is possible that recollection is affected quantitatively, meaning that individuals with ASD would exhibit more failures of recollection and a difficulty reconstructing the same amount of information from memory. In neurotypical adults, the success of recollection and the precision with which information is recollected have recently been associated with separable roles for the hippocampus and posterior parietal cortex, respectively (Richter, Cooper, et al., 2016), providing evidence of a dissociation in recollective-retrieval properties on the neurocognitive level.

**Fig. 2:**
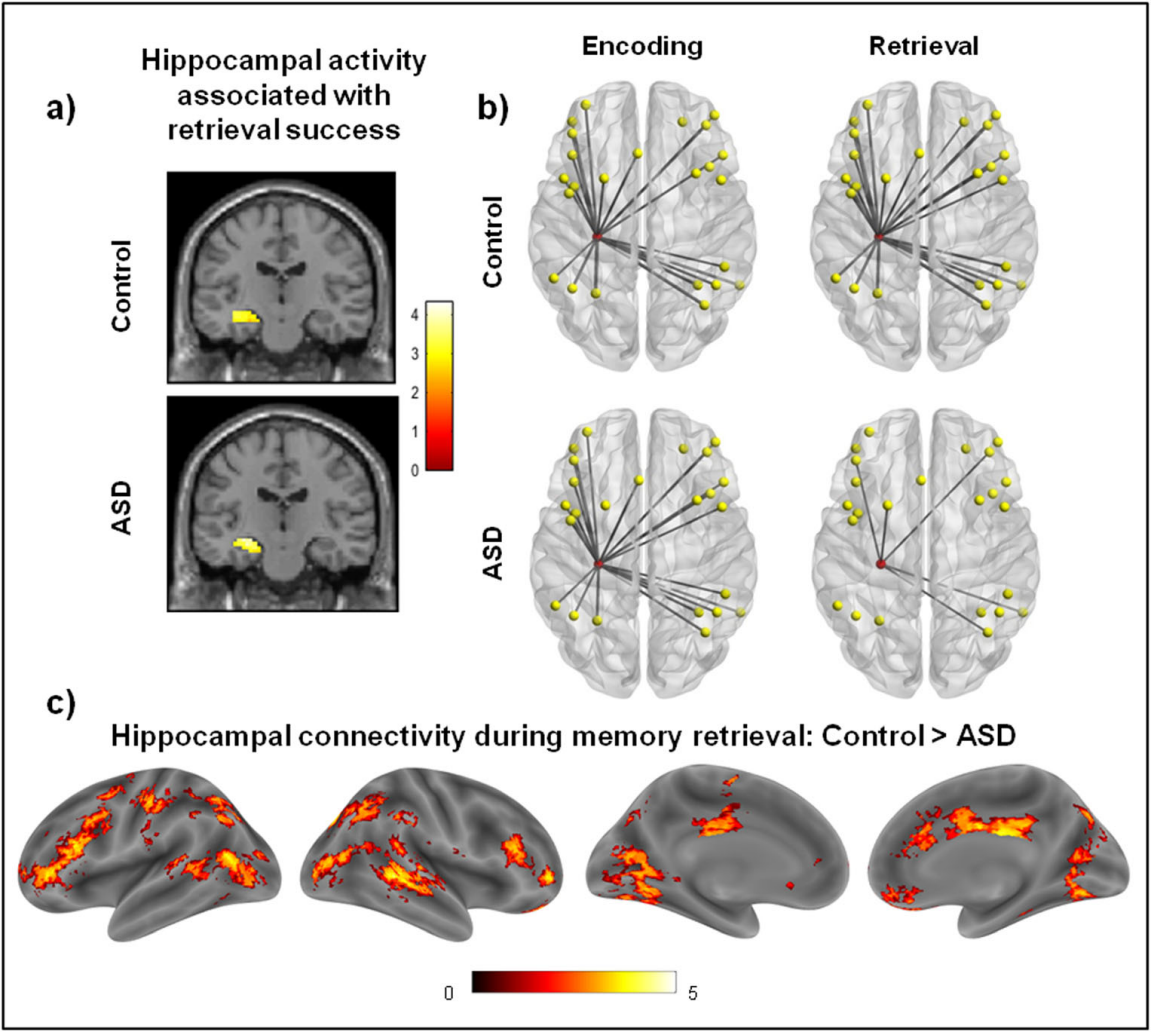
Figure adapted from Cooper et al. (2017b). (**a**) Participants with ASD and neurotypical controls exhibited similar hippocampal activity during successful recollection. (**b** and **c**) In contrast, widespread reductions in hippocampal functional connectivity (node in red) were observed in the ASD group relative to the control group during memory retrieval, particularly with regions of the fronto-parietal control network (nodes in yellow), but differences in connectivity strength were not observed during memory encoding. Effects are displayed at a threshold of *p* < .01 for visualization. Scales reflect *t* values

In the first fMRI study to assess recollection-based retrieval in ASD (Cooper et al., 2017b), adults with ASD were asked to re-create the appearance of objects presented on a scene background, facilitating separate estimates of retrieval success and retrieval precision (e.g., Bays et al., 2009; Brady et al., 2013; Harlow & Yonelinas, 2016). Participants with ASD exhibited a reduction in instances of recollection success but there was no evidence for an additional reduction in the precision of successfully retrieved memories. Neurally, comparable patterns of activity and functional connectivity were observed during memory encoding between the groups, but lateral prefrontal activity during encoding predicted subsequent memory only in the control group and not in the ASD group, mirroring the findings of Cooper et al.’s (2017a) previous eye-tracking study. Despite some evidence for attenuated lateral prefrontal activity during memory retrieval in the ASD participants, both groups showed comparable patterns of hippocampal, medial prefrontal, and posterior parietal activity during recollection. However, the ASD group exhibited substantially attenuated hippocampal functional connectivity during memory retrieval, particularly with regions of the fronto-parietal control network, but also with regions including middle temporal gyrus, middle cingulate gyrus, and caudate (see Fig. 2). These findings therefore reveal a novel dissociation between typical memory-related regional activity and reduced functional connectivity in ASD during memory retrieval, alongside no significant differences in brain function during memory encoding.

Based on findings regarding hippocampal activity and hippocampal connectivity during recollection within the neurotypical literature, as considered earlier, we suggest that memory representations may be processed and activated by the hippocampus in a similar manner in people with ASD and neurotypical individuals, but are not consciously reconstructed effectively during memory retrieval in ASD, primarily as a result of disrupted hippocampal connectivity (cf. Cabeza & Moscovitch, 2013; Simons & Spiers, 2003). Therefore, task-specific reductions in functional connectivity, rather than region-specific dysfunction, within the memory retrieval network in ASD may result in a difficulty explicitly reconstructing and recollecting past experiences, even in instances where elements of memory representations themselves are “intact.” This seems a likely possibility given the role of hippocampal-prefrontal connectivity in explicit recollection (Bowman & Dennis, 2016; Hannula & Ranganath, 2009) over and above hippocampal relational processes, and the importance of whole-brain hippocampal connectivity in facilitating episodic recollection (Geib et al., 2015; Schedlbauer et al., 2014). A fundamental difficulty in integrating memory representations with higher-order retrieval processes, resulting in attenuated explicit recollection of events, fits well with the already established idea that cognitive processing and memory in ASD is “heavily influenced by the here-and-now” (Bowler et al. 2011, p. 329), which could imply that individuals with ASD have difficulty reconstructing a memory or internal representation distinct from their immediate external environment. This characterization of episodic recollection in ASD is by no means entirely distinct from the ideas proposed in previous theoretical accounts. Rather, we believe that recent evidence points to a separable impairment in the *process* of recollective retrieval and we highlight the interactive neural network mechanisms which might contribute to such differences in memory in ASD.

These recent observations concerning the neurocognitive basis of recollection in ASD highlight how studying memory in this population has the potential to provide a fresh perspective on neurotypical memory. Traditional neuropsychology research has focused on determining how memory might be affected by dysfunction of individual brain regions such as the hippocampus or posterior parietal cortex, now considered to be nodes of large-scale functional brain networks involved in recollection. Studying such patients provides a wealth of information about the contribution of individual regions, but little insight into the interactions between them that appear to play such an important role. As a population that may be characterized more by prominent differences in inter-region connectivity than region-specific dysfunction within the memory retrieval network, ASD provides the opportunity to establish the cognitive functions associated with region-to-region or network interactions, complementing insights about individual regions gained from studying patients with selective hippocampal or parietal lesions, for example. Investigating ASD as a disorder of functional dysconnectivity, alongside traditional region-based neuropsychology, can provide unique information to researchers interested in neurotypical memory, especially given the recent rise in research studying how whole-brain network dynamics underpin episodic memory retrieval (e.g., Geib et al., 2015; King et al., 2015; Ritchey et al., 2015; Schedlbauer et al., 2014). However, much further neuroimaging work is needed to specify the neural basis of altered recollection in ASD before this new approach can be fully integrated with our understanding of episodic recollection more generally. In particular, a number of questions arise from the literature reviewed here concerning recollection dysfunction in ASD that will be important for future research to explore in order to further specify an integrative framework of the cognitive and neural processes involved in recollection experience in this population. We present some ideas below, which we hope might help to stimulate future investigations in this area.

## Future directions

To further investigate the influence of functional dysconnectivity on recollective experience in ASD, it is important to consider and investigate differences in connectivity strength in light of shifts in demands on recollection. Research within the neurotypical population suggests that dynamic changes in functional connectivity provide the task-specific brain state necessary to facilitate episodic memory retrieval. The notion of “process-specific alliances” was formalized by the component process model (Moscovitch, 1992; Cabeza & Moscovitch, 2013; Moscovitch et al., 2016) and describes how subsets of brain regions form transient alliances (connections) to facilitate task-specific cognitive processes that contribute to episodic memory retrieval. Thus, it is necessary to examine how connectivity alters (over and above neural activity) in response to changes in memory retrieval demands in ASD, from recognition to cued recall and free recall of complex events, for example. Given the poor temporal resolution of fMRI, however, it is difficult to conclude at which stage of the recollective process that reduced connectivity is most pronounced and whether pre-retrieval or post-retrieval processes may be more responsible for differences in recollection. Investigating the neural processes of episodic memory retrieval in ASD via EEG in addition to fMRI may provide additional insight to address this important question. Only two such studies have been conducted in ASD, reporting evidence for attenuated frontal old-new effects during both early and late stages of recognition memory trials (Massand et al., 2013) and time non-specific old-new effects in ASD compared to controls (Massand & Bowler, 2012). Such multi-modal neuroimaging research (cf. Bergstrom et al., 2013) is likely to provide valuable insight into the neurocognitive process-specific alliances that give rise to altered episodic recollection in people with ASD.

An important point to emphasize is that encoding and retrieval are highly interactive processes, meaning that any separable recollection difficulty is likely to affect encoding of new information. This distinction forms an important contrast between learning novel, experimental stimuli versus encoding situations in everyday life that are likely to be highly overlapping with previous episodic memories. Reinstatement of related past experiences forms a substantial part of how we interpret, organize, and consolidate new information (Mack & Preston, 2016), with this ability guided by medial prefrontal-hippocampal interactions (Schlichting & Preston, 2015). Therefore, if memory reinstatement occurs less frequently or automatically in people with ASD, then past memories may be less likely to influence how new memories are formed, consolidated, and integrated with existing knowledge. Although not directly researched, some evidence for this effect in ASD comes from studies of transitive inference, in which adults with ASD have been observed to show intact associative learning but a reduced ability to generalize these memories to novel associations compared to a control group (Solomon et al., 2011). Furthermore, people with ASD appear to be less able to generalize learning from one context to another and are less likely to use past experiences to influence how novel stimuli are categorized (Froehlich et al., 2012). Therefore, a key question concerns how episodic memories are integrated and change over time in ASD, rather than just how single episodic memories are formed and retrieved. The application of multivoxel pattern analysis (MVPA) to fMRI data allows researchers to gain a degree of insight into the representations of individual memories (Rissman & Wagner, 2012) and could, therefore, provide valuable information about recollection in ASD. In particular, MVPA could provide further evidence concerning the specificity of encoded representations, as well as whether encoding is less affected by reinstatement of previous experiences in people with ASD, and whether memories are reinstated automatically during memory retrieval but cannot be explicitly retrieved, or whether a reduction in recollection is reflected in impaired ability to neurally reinstate previous experiences.

An additional modulatory effect on recollection that needs to be more thoroughly investigated and incorporated into a neurocognitive account of recollection in ASD is emotion. The research discussed here has focused on recollection of non-emotional information in ASD, but emotion, particularly negative emotion, is known to have a distinct effect on recollection-based memory retrieval in the neurotypical population by increasing memory for item details but often diminishing memory for contextual information (Kensinger, 2009). Interestingly, although the evidence is somewhat mixed, there are observations that memory in ASD may be less moderated by emotion compared to typical individuals (Deurelle et al., 2008; Gaigg & Bowler, 2009). For example, Gaigg and Bowler (2008) observed the typical increase in immediate recall of emotional and semantic words in ASD as seen in controls but, in the ASD group, emotional words were not as resistant to forgetting as in typical individuals. Thus, it is possible that an atypical influence of emotion on recollection in ASD may result from altered consolidation of item-emotion bindings mediated by the amygdala (Yonelinas & Ritchey, 2015). However, two studies have also identified typical emotional benefits on memory in ASD (cf. Maras et al., 2012; South et al., 2008), with Maras et al. showing this effect for naturalistic events. It remains unclear, therefore, if and how factors that modulate memory consolidation in neurotypical individuals operate atypically in ASD. Further research should be conducted in this area before emotion can be integrated with the pattern of episodic recollection for neutral information observed in this population.

Another important line of research in order to address the basis of altered recollection is to establish the relationship between memory functioning and other core characteristics of ASD, such as differences in social functioning and flexibility of behavior. Such relationships are yet to be thoroughly investigated but will prove to be an important avenue of future research if episodic memory is to be embedded within a broader neurocognitive theory of ASD. As such, it is important for research to explain the trajectory of episodic memory development in ASD and how the neurocognitive mechanisms we observe to function atypically in adults emerge through childhood and adolescence. For instance, Perner et al. (2007) have argued that development of episodic memory is closely linked to development of theory of mind, but it is unclear how dependent these processes are on one another in ASD. Interestingly, Bachevalier and Loveland (2006) have argued that the development of frontal-medial temporal networks in ASD underpins the heterogeneity of socio-emotional processes and memory that is observed, which fits well with the proposal here that differences in episodic recollection are likely underpinned by altered communication between medial temporal and fronto-parietal regions.

Relatedly, the current discussion has focused on research in individuals without concurrent language or learning difficulties because the vast majority of memory studies, particularly those targeting recollection, in ASD have involved such participants. Boucher et al. (2012), in their comprehensive review of memory functioning across the autism spectrum, aimed to provide insight into possible differences in mnemonic processes as a function of co-occurring language and intellectual impairments, where difficulties with familiarity-based recognition memory may map on to differences in language ability across the spectrum, for example. However, despite efforts to develop equivalent memory paradigms to account for heterogeneity in the ASD population (cf. Bigham et al., 2010), it is very difficult to control for impairments in other cognitive domains involved in memory tasks. This is particularly the case for experiments targeting complex episodic memories, which rely heavily on introspection, language, and executive function. It is thus largely unknown how the *subjective experience* of recollection differs across the autism spectrum. In order to address this gap in the literature, future research should aim to develop tasks that involve participants encoding and subsequently reconstructing (cf. Cooper et al., 2017b) or identifying specific details of naturalistic past events in their own time, with different levels of retrieval cue. Such tasks may be able to reveal systematic changes in the content and quality of recollective experience while minimizing the influence of any language or cognitive control difficulties.

In summary, we suggest that current theoretical accounts of episodic memory in ASD may not fully capture the nature of recollection difficulties in this population. Moreover, the current literature on recollection in ASD has often been inconsistent, with little continuity of methods, clear definitions of terminology, or systematic ways of testing theoretical approaches and distinguishing between distinct encoding and retrieval processes. We argue that recent research suggests that people with ASD exhibit a distinct impairment in the explicit process of recollective retrieval, which is likely to be best understood in terms of altered functional connectivity between core regions of the memory retrieval network, particularly with hubs such as the hippocampus. Therefore, an integrative approach, focusing on interactions between cognitive processes and neural networks, will provide a useful framework for studying episodic recollection in the disorder. This neurocognitive profile means that ASD may provide valuable insights into the recollection processes supported by specific functional interactions in the neurotypical brain. We have also proposed several lines of investigation for future ASD research to explore. Firstly, it is important for research to specify the effects of aberrant retrieval-related connectivity on recollection in ASD and to investigate how episodic memories are represented and change over time on the neural level. Secondly, recollection differences should be compared and related to the development of other cognitive and neural functions in ASD, with particular consideration of differences between individuals with and without additional language and intellectual impairments. Such research will help us to further specify and understand the unique neurocognitive basis of episodic recollection in people with ASD.

### Author Note

RAC was funded by the Economic and Social Research Council and JSS by the James S. McDonnell Foundation. The work was supported by the University of Cambridge Behavioural and Clinical Neuroscience Institute, funded by a joint award from the Medical Research Council and the Wellcome Trust.

## References

[CR1] Addis DR, McAndrews MP (2006). Prefrontal and hippocampal contributions to the generation and binding of semantic associations during successful encoding. NeuroImage.

[CR2] Aggleton JP, Sanderson DJ, Pearce JM (2007). Structural learning and the hippocampus. Hippocampus..

[CR3] Awipi T, Davachi L (2008). Content-specific source encoding in the human medial temporal lobe. Journal of Experiment Psychology: Learning, Memory,and Cognition.

[CR4] Bachevalier J, Loveland KA (2006). The orbitofrontal-amygdala circuit and self-regulation of social-emotional behavior in autism. Neuroscience and Biobehavioral Reviews.

[CR5] Badre D, Wagner AD (2007). Left ventrolateral prefrontal cortex and the cognitive control of memory. Neuropsychologia.

[CR6] Barttfeld P, Wicker B, Cukier S, Navarta S, Lew S, Leiguarda R, Sigman M (2012). State-dependent changes of connectivity patterns and functional network topology in autism spectrum disorder. Neuropsychologia.

[CR7] Bays PM, Catalao RF, Husain M (2009). The precision of visual working memory is set by allocation of a shared resource. Journal of Vision..

[CR8] Benoit RG, Schacter DL (2015). Specifying the core network supporting episodic simulation and episodic memory by activation likelihood estimation. Neuropsychologia.

[CR9] Bennetto L, Pennington B, Rogers S (1996). Intact and impaired memory functions in autism. Child Development.

[CR10] Ben Shalom D (2009). The medial prefrontal cortex and integration in autism. The Neuroscientist : A Review Journal Bringing Neurobiology, Neurology and Psychiatry.

[CR11] Bergstrom ZM, Henson RN, Taylor JR, Simons JS (2013). Multimodal imaging reveals the spatiotemporal dynamics of recollection. NeuroImage.

[CR12] Beversdorf DQ, Anderson JM, Manning SE, Anderson SL, Nordgren RE, Felopulos GJ, Nadeau SE, Heilman KM, Bauman ML (1998). The effect of semantic and emotional context on written recall for verbal language in high functioning adults with autism spectrum disorder. Journal of Neurology, Neurosurgery, & Psychiatry.

[CR13] Beversdorf DQ, Smith BW, Crucian GP, Anderson JM, Keillor JM, Barrett AM, Hughes JD, Felopulos GJ, Bauman ML, Nadeau SE, Heilman KM (2000). Increased discrimination of “false memories” in autism spectrum disorder. PNAS.

[CR14] Bigham S, Boucher J, Mayes A, Anns S (2010). Assessing recollection and familiarity in autistic spectrum disorders: methods and findings. Journal of Autism and Developmental Disorders.

[CR15] Blumenfeld RS, Ranganath C (2007). Prefrontal cortex and long-term memory encoding: An integrative review of findings from neuropsychology and neuroimaging. The Neuroscientist.

[CR16] Bonnici HM, Richter FR, Yazar Y, Simons JS (2016). Multimodal feature integration in the angular gyrus during episodic and semantic retrieval. The Journal of Neuroscience.

[CR17] Boucher J (2007). Memory and generativity in very high functioning autism. Autism.

[CR18] Boucher J, Bigham S, Mayes A, Muskett T (2008). Recognition and language in low functioning autism. Journal of Autism and Developmental Disorders.

[CR19] Boucher J, Mayes A, Bigham S (2012). Memory in autistic spectrum disorder. Psychological Bulletin.

[CR20] Boucher J, Mayes A (2012). Memory in ASD: have we been barking up the wrong tree?. Autism.

[CR21] Boucher J, Warrington EK (1976). Memory deficits in early infantile autism: some similarities to the amnesic syndrome. British Journal of Psychology.

[CR22] Bowler DM, Gaigg SB, Gardiner JM (2008). Effects of related and unrelated context on recall and recognition by adults with high-functioning autism spectrum disorder. Neuropsychologia.

[CR23] Bowler DM, Gaigg SB, Gardiner JM (2009). Free recall learning of hierarchically organised lists by adults with Asperger’s syndrome: additional evidence for dimished relational processing. Journal of Autism and Developmental Disorders.

[CR24] Bowler DM, Gaigg SB, Gardiner JM (2010). Multiple list learning in adults with autism spectrum disorder: parallels with frontal lobe damage or further evidence of diminshed relational processing?. Journal of Autism and Developmental Disorders.

[CR25] Bowler DM, Gaigg SB, Gardiner JM (2014). Binding of Multiple Features in Memory by High-Functioning Adults with Autism Spectrum Disorder. Journal of Autism and Developmental Disorders.

[CR26] Bowler DM, Gaigg SB, Gardiner JM (2015). Brief Report: The Role of Task Support in the Spatial and Temporal Source Memory of Adults with Autism Spectrum Disorder. Journal of Autism and Developmental Disorders.

[CR27] Bowler, D. M., Gaigg, S. B., & Lind, S. E. (2011). Memory in Autism : binding , self and brain. In *Researching the autism spectrum: contemporary perspectives* (pp. 316–346). Cambridge: Cambridge University Press.

[CR28] Bowler DM, Gardiner JM, Berthollier N (2004). Source Memory in Adolescents and Adults with Asperger’s Syndrome. Journal of Autism and Developmental Disorders.

[CR29] Bowler DM, Gardiner JM, Gaigg SB (2007). Factors affecting conscious awareness in the recollective experience of adults with Asperger’s syndrome. Consciousness and Cognition.

[CR30] Bowler DM, Gardiner JM, Grice SJ (2000). Episodic memory and remembering in adults with Asperger syndrome. Journal of Autism and Developmental Disorders.

[CR31] Bowler DM, Matthews NJ, Gardiner JM (1997). Asperger’s syndrime and memory: similarity to autism but not amnesia. Neuropsychologia.

[CR32] Bowman CR, Dennis NA (2016). The neural basis of recollection rejection: increases in hippocampal-prefrontal connectivity in the absence of a shared recall-to-reject and target recollection network. Journal of Cognitive Neuroscience.

[CR33] Brady TF, Konkle T, Gill J, Oliva A, Alvarez GA (2013). Visual long-term memory has the same limit on fidelity as visual working memory. Psychological Science.

[CR34] Brezis RS (2015). Memory integration in the autobiographical narratives of individuals with autism. Frontiers in Human Neuroscience.

[CR35] Brezis RS, Galili T, Wong T, Piggot JI (2014). Impaired Social Processing in Autism and its Reflections in Memory: A Deeper View of Encoding and Retrieval Processes. Journal of Autism and Developmental Disorders.

[CR36] Bruck M, London K, Landa R, Goodman J (2007). Autobiographical memory and suggestibility in children with autism spectrum disorder. Development and Psychopathology.

[CR37] Buckner RL, Carroll DC (2007). Self-projection and the brain. Trends in Cognitive Sciences.

[CR38] Cabeza R, Moscovitch M (2013). Memory systems, processing modes, and components: functional neuroimaging evidence. Perspectives in Psychological Science.

[CR39] Carmo JC, Duarte E, Pinho S, Filipe CN, Marques JF (2016). Preserved proactive interference in autism spectrum disorder. Journal of Autism and Developmental Disorders.

[CR40] Ciaramelli E, Faggi G, Scarpazza C, Mattioli F, Spaniol J, Ghetti S, Moscovitch M (2017). Subjective recollection independent from multifeatural context retrieval following damage to the posterior parietal cortex. Cortex.

[CR41] Cooper RA, Plaisted-Grant KC, Baron-Cohen S, Simons JS (2016). Reality monitoring and metamemory in adults with autism spectrum conditions. Journal of Autism and Developmental Disorders.

[CR42] Cooper RA, Plaisted-Grant KC, Baron-Cohen S, Simons JS (2017). Eye movements reveal a dissociation between memory encoding and retrieval in adults with autism. Cognition.

[CR43] Cooper RA, Plaisted-Grant KC, Hannula DE, Ranganath C, Baron-Cohen S, Simons JS (2015). Impaired recollection of visual scene details in adults with autism spectrum conditions. Journal of Abnormal Psychology.

[CR44] Cooper RA, Richter FR, Bays PM, Plaisted-Grant KC, Baron-Cohen S, Simons JS (2017). Reduced hippocampal functional connectivity during episodic memory retrieval in autism. Cerebral Cortex.

[CR45] Courchesne E, Pierce K (2005). Why the frontal cortex in autism might be talking only to itself: local over-connectivity but long-distance disconnection. Current Opinion in Neurobiology.

[CR46] Cowell RA, Bussey TJ, Saksida LM (2010). Components of recognition memory: dissociable cognitive processes of just differences in representational complexity?. Hippocampus.

[CR47] Crane L, Goddard L (2008). Episodic and semantic autobiographical memory in adults with autism spectrum disorders. Journal of Autism and Developmental Disorders.

[CR48] Crane L, Goddard L, Pring L (2009). Specific and general autobiographical knowledge in adults with autism spectrum disorders: The role of personal goals. Memory.

[CR49] Crane L, Lind SE, Bowler DM (2013). Remembering the past and imaging the future in autism spectrum disorder. Memory.

[CR50] Crane L, Pring L, Jukes K, Goddard L (2012). Patterns of autobiographical memory in adults with autism spectrum disorder. Journal of Autism and Developmental Disorders.

[CR51] Damarla SR, Keller TA, Kana RK, Cherkassky VL, Williams DL, Minshew NJ, Just MA (2010). Cortical underconnectivity coupled with preserved visuospatial cognition in autism: Evidence from an fMRI study on an embedded figures task. Autism Research.

[CR52] Davachi L (2006). Item, context and relational episodic encoding in humans. Current Opinion in Neurobiology.

[CR53] Davidson, P. S. R., Anaki, D., Ciaramelli, E., Cohn, M., Kim, A. S. N., Murphy, K. J., Troyer, A. K., Moscovitch, M., & Levine, B. (2008). Does lateral parietal cortex support episodic memory? Evidence from focal lesion patients. *Neuropsychologia, 46(7),* 1743-1755. 10.1016/j.neuropsychologia.2008.01.011 PMC2806230 18313699

[CR54] Demb JB, Desmond JE, Wagner AD, Vaidya CJ, Glover GH, Gabrieli JD (1995). Semantic encoding and retrieval in the left inerior preforntal cortex: a functional MRI study of task difficulty and prcoess specificity. Journal of Neuroscience.

[CR55] Dennis NA, Turney IC, Webb CE, Overman AA (2015). The effects of item familiarity on the neural correlates of successful associative memory encoding. Cognitive, Affective, and Behavioral Neuroscience.

[CR56] Deurelle C, Hubert B, Santos A, Wicker B (2008). Negative emotion does not enhance recall skils in adults with autistic spectrum disorders. Autism Research.

[CR57] Dew ITZ, Cabeza R (2011). The porous boundaries betwen explicit and implicit memory: behavioral and neural evidence. Ann. N.Y. Acad. Sci.

[CR58] Diana RA, Yonelinas AP, Ranganath C (2007). Imaging recollection and familiarity in the medial temporal lobe: a three-component model. Trends in Cognitive Sciences.

[CR59] Dobbins IG, Foley H, Schacter DL, Wagner AD (2002). Executive control during episodic retrieval: multiple prefrontal processes subserve source memory. Neuron.

[CR60] Drowos DB, Berryhill M, Andre JM, Olson IR (2010). True memory, false memory, and subjective recollection deficits after focal parietal lobe lesions. Neuropsychology.

[CR61] Duss SB, Reber TP, Hanggi J, Schwab S, Wiest R, Muri RM, Brugger P, Gutbroad K, Henke K (2014). Unconscious relational encoding depends on hippocampus. Brain.

[CR62] Eichenbaum H, Amaral DG, Buffalo EA, Buzsaki G, Cohen N, Davachi L, Frank L, Heckers S, Morris RGM, Moser EI, Nadel L, O’Keefe J, Preston A, Ranganath C, Silva A, Witter M (2016). Hippocampus at 25. Hippocampus.

[CR63] Eichenbaum H, Yonelinas AR, Ranganath C (2007). The medial temporal lobe and recognition memory. Annual Review of Neuroscience.

[CR64] Elfman KW, Yonelinas AP (2015). Recollection and familiarity exhibit dissociable similarity gradients: a test of the complementary learning systems model. Journal of Cognitive Neuroscience.

[CR65] Farrant A, Blades M, Boucher J (1998). Source monitoring by children with autism. Journal of Autism and Developmental Disorders.

[CR66] Ford JH, Kensinger EA (2016). Effects of internal and external vividness on hippocampal connectivity during memory retrieval. Neurobiology of Learning and Memory.

[CR67] Fornito A, Harrison BJ, Zalesky A, Simons JS (2012). Competitive and cooperative dynamics of large-scale brain functional networks supporting recollection. PNAS.

[CR68] Froelich AL, Anderson JS, Bigler ED, Miller JS, Lange NT, DuBray MB, Cooperrider JR, Cariello A, Nielsen JA, Lainhart JE (2012). Intact prototype formation but impaired generalization in autism. Research in Autism Spectrum Disorder.

[CR69] Gaigg SB, Bowler DM (2008). Free recall and forgetting of emotionally arousing words in autism spectrum disorder. Neuropsychologia.

[CR70] Gaigg SB, Bowler DM (2009). Illusory memories of emotionally charged words in autism spectrum disorder further evidence for atypical emotion processing outside the social domain. Journal of Autism and Developmental Disorders.

[CR71] Gaigg SB, Bowler DM, Ecker C, Calvo-Merino B, Murphy DG (2015). Episodic recollection difficulties in ASD result from atypical relational encoding: behavioral and neural evidence. Autism Research.

[CR72] Gaigg SB, Bowler DM, Gardiner JM (2014). Episodic but not semantic order memory difficulties in autism spectrum disorder: evidence from the historical figures task. Memory.

[CR73] Gaigg SB, Gardiner JM, Bowler DM (2008). Free recall in autism spectrum disorder: the role of relational and item-specific encoding. Neuropsychologia.

[CR74] Gallo DA, McDonough IM, Scimeca J (2010). Dissociating source memory decisions in the prefrontal cortex: fMRI of diagnostic and disqualifying monitoring. Journal of Cognitive Neuroscience.

[CR75] Gardiner JM, Bowler DM, Grice SJ (2003). Further evidence of preserved priming and impaired recall in adults with Asperger’s syndrome. Journal of Autism and Developmental Disorders.

[CR76] Gardiner JM, Gregg VH, Mashru R, Thaman M (2001). Impact of encoding depth on awareness of perceptual effects in recognition memory. Memory & Cognition.

[CR77] Geib BR, Stanley ML, Wing EA, Laurienti PJ, Cabeza R (2015). Hippocampal contributions to the large-scale episodic memory network predict vivid visual memories. Cerebral Cortex.

[CR78] Goddard L, Dritschel B, Robinson S, Howlin P (2014). Development of autobiographical memory in children with autism spectrum disorders: Deficits, gains, and predictors of performance. Development and Psychopathology.

[CR79] Goddard L, Howlin P, Dritschel B, Patel T (2007). Autobiographical memory and social problem-solving in asperger syndrome. Journal of Autism and Developmental Disorders.

[CR80] Gordon AM, Rissman J, Kiani R, Wagner AD (2014). Cortcial reinstatement mediates the relationship between content-specific encoding activity and subsequent recollection decisions. Cerebral Cortex.

[CR81] Grainger C, Williams DM, Lind SE (2014). Online action monitoring and memory for self-performed actions in autism spectrum disorder. Journal of Autism and Developmental Disorders.

[CR82] Grainger C, Williams DM, Lind SE (2014). Metacognition, metamemory, and mindreading in high-functioning adults with autism spectrum disorder. Journal of Abnormal Psychology.

[CR83] Grainger C, Williams DM, Lind SE (2016). Judgment of learning accuracy in high-functioning adolescents and adults with autism spectrum disorder. Journal of Autism and Developmental Disorders.

[CR84] Green DM, Swets JA (1966). Signal detection theory and psychophysics.

[CR85] Greimel E, Nehrkorn B, Fink GR, Kukolja J, Kohls G, Müller K, Schulte-Rüther M (2012). Neural mechanisms of encoding social and non-social context information in autism spectrum disorder. Neuropsychologia.

[CR86] Grisdale E, Lind SE, Eacott MJ, Williams DM (2014). Self-referential memory in autism spectrum disorder and typical development: Exploring the ownership effect. Consciousness & Cognition.

[CR87] Hala S, Rasmussen C, Henderson AME (2005). Three types of source monitoring by children with and without autism: the role of executive function. Journal of Autism and Developmental Disorders.

[CR88] Hannula DE, Greene AJ (2012). The hippocampus reevaluated in unconscious learning and memory: at a tipping point?. Frontiers in Human Neuroscience.

[CR89] Hannula DE, Ranganath C (2009). The Eyes Have It: Hippocampal Activity Predicts Expression of Memory in Eye Movements. Neuron.

[CR90] Hannula DE, Ranganath C, Ramsay IS, Solomon M, Yoon J, Niendam TA, Carter CS, Ragland JD (2010). Use of eye movement monitoring to examine item and relational memory in schizophrenia. Biological Psychiatry.

[CR91] Hannula DE, Tranel D, Allen JS, Kirchhoff BA, Nickel AE, Cohen NJ (2015). Memory for items and relationships among items embedded in realistic scenes: disproportionate relational memory impairments in amnesia. Neuropsychology.

[CR92] Harlow IM, Yonelinas AP (2016). Distinguishing between the success and precision of recollection. Memory.

[CR93] Hedley D, Young R, Brewer N (2012). Using eye movements as an index of implicit face recognition in autism spectrum disorder. Autism Research.

[CR94] Henderson HA, Zahka NE, Kojkowski NM, Inge AP, Schwartz CB, Hileman CM, Coman DC, Mundy PC (2009). Self-referenced memory, social cognition, and symptom presentation in autism. Journal of Child Psychology and Psychiatry.

[CR95] Hill EL (2004). Executive dysfunction in autism. Trends in Cognitive Sciences.

[CR96] Hill EL, Russell J (2002). Action memory and self-monitoring in children with autism : self versus other. Infant and Child Development.

[CR97] Horner AJ, Bisby JA, Bush D, Lin W-J, Burguess N (2015). Evidence for holistic episodic recollection via hippocampal pattern completion. Nature Communications.

[CR98] Hower KH, Wixted J, Berryhill ME, Olson IR (2014). Impaired perception of mnemonic oldness, but not mnemonic newness, after parietal lobe damage. Neuropsychologia.

[CR99] Hunt RR, Seta CE (1984). Category size effects in recall: The roles of relational and item information. Journal of Experimental Psychology: Learning, Memory, and Cognition.

[CR100] Johnson M, Hashtroudi S, Lindsay D (1993). Source monitoring. Psychological Bulletin.

[CR101] Just MA, Keller TA, Malave VL, Kana RK, Varma S (2012). Autism as a neural systems disorder: A theory of frontal-posterior underconnectivity. Neuroscience & Biobehavioural Reviews.

[CR102] Kafkas A, Montaldi D (2011). Recognition memory strength is predicted by pupillary responses at encoding while fixation patterns distinguish recollection from familiarity. Quarterly Journal of Experimental Psychology.

[CR103] Kamio Y, Toichi M (2007). Memory illusion in high-functioning autism and Asperger’s disorder. Journal of Autism and Developmental Disorders.

[CR104] Kana RK, Libero LE, Moore MS (2011). Disrupted cortical connectivity theory as an explanatory model for autism spectrum disorders. Physics of Life Reviews.

[CR105] Kennedy DP, Courchesne E (2008). Functional abnormalities of the default network during self- and other-reflection in autism. Social Cognitive and Affective Neuroscience.

[CR106] Kensinger EA (2009). Remembering the details: Effects of emotion. Emotion Review: Journal of the International Society for Research on Emotion.

[CR107] Kim H (2016). Default network activation during episodic and semantic memory retrieval: a selective meta-analytic comparison. Neuropsychologia.

[CR108] King DR, de Chastelaine M, Elward RL, Wang TH, Rugg MD (2015). Recollection-related increases in functional connectivity predict individual differences in memory accuracy. The Journal of Neuroscience.

[CR109] Konkel A, Cohen NJ (2009). Relational memory and the hippocampus: representations and methods. Frontiers in Neuroscience.

[CR110] Konkel A, Warren DE, Duff MC, Tranel DN, Cohen NJ (2008). Hippocampal amnesia impairs all manner of relational memory. Frontiers in Human Neuroscience.

[CR111] Koshino H, Kana RK, Keller TA, Cherkassky VL, Minshew NJ, Just MA (2008). fMRI investigation of working memory for faces in autism: visual coding and underconnectivity with frontal areas. Cerebral Cortex.

[CR112] Kuhl BA, Chun MM (2014). Successful remembering elicits event-specific activity patterns in lateral parietal cortex. The Journal of Neuroscience.

[CR113] Laeng B, Bloem IM, D’ASDenzo S, Tommasi L (2014). Scrutinizing visual images: the role of gaze in mental imagery and memory. Cognition.

[CR114] Leshikar ED, Duarte A (2012). Medial prefrontal cortex supports source memory accuracy for self-referenced items. Social Neuroscience.

[CR115] Lind SE (2010). Memory and the self in autism: A review and theoretical framework. Autism : The International Journal of Research and Practice.

[CR116] Lind SE, Bowler DM (2009). Recognition memory, self-other source memory, and theory-of-mind in children with autism spectrum disorder. Journal of Autism and Developmental Disorders.

[CR117] Lind SE, Bowler DM (2010). Episodic Memory and Episodic Future Thinking in Adults With Autism. Journal of Abnormal Psychology.

[CR118] Lind SE, Williams DM, Bowler DM, Peel A, Raber J (2014). Episodic memory and episodic future thinking impairments in high-functioning autism spectrum disorder: An underlying difficulty with scene construction or self-projection?. Neuropsychology.

[CR119] Lind S, Williams D, Raber J, Peel A, Bowler DM (2013). Spatial navigation impairments among intellectually high-functioning adults with autism spectrum disorder: Exploring relations with theory of mind, episodic memory, and episodic future thinking. Journal of Abnormal Psychology.

[CR120] Liu Z-X, Shen K, Olsen RK, Ryan JD (2017). Visual sampling predicts hippocampal activity. Journal of Neuroscience.

[CR121] Lombardo MV, Baron-Cohen S (2011). The role of the self in mindblindness in autism. Consciousness and Cognition.

[CR122] Lombardo MV, Barnes JL, Wheelwright SJ, Baron-Cohen S (2007). Self-referential cognition and empathy in autism. PloS One.

[CR123] Lombardo MV, Chakrabati B, Bullmore ET, Sadek SA, Pasco G, Wheelwright SJ, Suckling J, Consortium MRCAIMS, Baron-Cohen S (2009). Atypical neural self-representation in autism. Brain.

[CR124] Loth E, Carlos Gómez J, Happé F (2011). Do high-functioning people with autism spectrum disorder spontaneously use event knowledge to selectively attend to and remember context-relevant aspects in scenes?. Journal of Autism and Developmental Disorders.

[CR125] Mack ML, Preston AR (2016). Decisions abut the past are guided by reinstatement of specific memories in the hippocampus and perirhinal cortex. NeuroImage.

[CR126] Maister L, Simons JS, Plaisted-Grant K (2013). Executive functions are employed to process episodic and relational memories in children with autism spectrum disorders. Neuropsychology.

[CR127] Maras K, Bowler DM (2011). Brief report: schema consistent misinformation effects in eyewitnesses with autism spectrum disorder. Journal of Autism and Developmental Disorders.

[CR128] Maras KL, Bowler DM (2012). Context reinstatement effects on eyewitness memory in autism spectrum disorder. British Journal of Psychology.

[CR129] Maras KL, Gaigg SB, Bowler DM (2012). Memory for emotionally arousing events over time in autism spectrum disorder. Emotion.

[CR130] Maras KL, Memon A, Lambrechts A, Bowler DM (2013). Recall of a live and personally experienced eyewitness event by adults with autism spectrum disorder. Journal of Autism and Developmental Disorders.

[CR131] Massand E, Bowler DM (2012). A typical neurophysiology underlying episodic and semantic memory in adults with autism spectrum disorder. Journal of Autism and Developmental Disorders.

[CR132] Massand E, Bowler DM, Mottron L, Hosein A, Jemel B (2013). ERP correlates of recognition memory in autism spectrum disorder. Journal of Autism and Developmental Disorders.

[CR133] McClelland JL, McNaughton BL, O’Reilly RC (1995). Why there are complementary learning systems in the hippocampus and neocortex: Insights from the successes and failure of connectionist models of learning and memory. Psychologial Review.

[CR134] Meyer BJ, Gardiner JM, Bowler DM (2014). Directed forgetting in high-functioning adults with autism spectrum disorders. Journal of Autism and Developmental Disorders.

[CR135] Minshew NJ, Goldstein G (2001). The Pattern of Intact and Impaired Memory Functions in Autism. Journal of Child Psychology and Psychiatry.

[CR136] Minshew NJ, Williams DL (2007). The new neurobiology of autism. Neurological Review.

[CR137] Molitor RJ, Ko PC, Hussey EP, Ally BA (2014). Memory-related eye movements challenge behavioral measures of pattern completion and pattern separation. Hippocampus.

[CR138] Morcom AM, Rugg MD (2012). Retrieval orientation and the control of recollection: an fMRI study. Journal of Cognitive Neuroscience.

[CR139] Moscovitch M (1992). Memory and working with memory: a component process model based on modules and central systems. Journal of Cognitive Neuroscience.

[CR140] Moscovitch M (2008). The hippocampus as a “stupid”, domain-specific module: Implications for theories of recent and remote memory, and of imagination. Canadian Journal of Experimental Psychology.

[CR141] Moscovitch M, Cabeza R, Winocur G, Nadel L (2016). Episodic memory and beyond: The hippocampus and neocortex in transformation. Annual Review of Psychology.

[CR142] Moss HE, Abdallah S, Fletcher P, Bright P, Pilgrim L, Acres K, Tyler LK (2005). Selecting among competing alternatives: selection and retrieval in the left inferior frontal gyrus. Cerebral Cortex.

[CR143] Mottron L, Morasse K, Belleville S (2001). A study of memory funtioning in individuals with autism. Journal of Child Psychology and Psychiatry.

[CR144] Mottron, L, Soulières, I., & Dawson, M. (2008). How useful are distinctions built for non-autistic in describing autistic memory? In J. Boucher & D. Bowler (Eds), Memory in autism. Cambridge University Press.

[CR145] Mullally SL, Maguire EA (2014). Memory, Imagination, and Predicting the Future: A Common Brain Mechanism?. The Neuroscientist.

[CR146] O’Shea A, Fein D, Cillessen A (2005). Source memory in children with autism spectrum disorders. Developmental Neuopsychology.

[CR147] Olsen RK, Chiew M, Buchsbaum BR, Ryan JD (2014). The relationship between delay period eye movements and visuospatial memory. Journal of Vision.

[CR148] Olsen RK, Moses SNM, Riggs L, Ryan JD (2012). The hippocampus supports multiple cognitive processes through relational binding and comparison. Frontiers in Human Neuroscience.

[CR149] Onyper SV, Zhang Y, Howard MW (2010). Some-or-none recollection: evidence from item and source memory. Journal of Experimental Psychology: General.

[CR150] Otten LJ (2007). Fragments of a larger whole: retrieval cues constrain observed neural correlates of memory encoding. Cerebral Cortex.

[CR151] Otten LJ, Henson RNA, Rugg MD (2001). Depth of processing effects on neural correlates of memory encoding: Relationship between findings from across- and within-task comparisons. Brain.

[CR152] Park H, Rugg MD (2011). Neural correlates of encoding within- and across-domain inter-item associations. Journal of Cognitive Neuroscience.

[CR153] Perner J, Kloo D, Gornik E (2007). Episodic memory development: theory of mind is part of re-experiencing experienced events. Infant and Child Development.

[CR154] Pertzov Y, Avidan G, Zohary E (2009). Accumulation of visual information across multiple fixations. Journal of Vision.

[CR155] Preston AR, Eichenbaum H (2013). Interplay of hippocampus and prefrontal cortex in memory. Current Biology.

[CR156] Ranganath C (2010). A unified framework for the functional organization of the medial temporal lobes and the phenomenology of episodic memory. Hippocampus.

[CR157] Ranganath C, Yonelinas AP, Cohen MX, Dy CJ, Tom SM, D’Esposito M (2003). Dissociable correlates of recollection and familiarity within the medial temporal lobes. Neuropsychologia.

[CR158] Reber TP, Luechinger R, Boesiger P, Henke K (2012). Unconscious relational inference recruits the hippocampus. The Journal of Neuroscience.

[CR159] Richter FR, Cooper RA, Bays PM, Simons JS (2016). Distinct neural mechanisms underlie the success, precision, and vividness of episodic memory. eLife.

[CR160] Ring, M., Bowler, D. M., & Gaigg, S. B. (2017a). An eye-movement study of relational memory in adults with autism spectrum disorder. *Journal of Autism and Developmental Disorders*, 10.1007/s10803-017-3212-3 10.1007/s10803-017-3212-3 PMC5602038 28688076

[CR161] Ring M, Derwent CLT, Gaigg SB, Bowler DM (2017). Structural learning difficulties implicate altered hippocampal functioning in adults with Autism Spectrum Disorder. Journal of Abnormal Psychology.

[CR162] Ring M, Gaigg SB, Bowler DM (2015). Object-location memory in adults with autism spectrum disorder. Autism Research.

[CR163] Ring M, Gaigg SB, Bowler DM (2016). Relational memory processes in adults with autism spectrum disorder. Autism Research.

[CR164] Rissman J, Wagner AD (2012). Distributed representations in memory: insights from functional brain imaging. Annual Review of Psychology.

[CR165] Ritchey M, Wing EA, LaBar KS, Cabeza R (2013). Neural Similarity Between Encoding and Retrieval is Related to Memory Via Hippocampal Interactions. Cerebral Cortex.

[CR166] Ritchey M, Libby LA, Ranganath C (2015). Cortico-hippocampal systems involved in memory and cognition: the PMAT framework. Prog Brain Res.

[CR167] Robin J, Hirshhorn M, Rosenbaum RS, Winocur G, Moscovitch M, Grady CL (2015). Functional connectivity of hippocampal and prefrontal networks during episodic and spatial memory based on real-world environments. Hippocampus.

[CR168] Rugg MD, Vilberg KL (2013). Brain networks underlying episodic memory retrieval. Current Opinion in Neurobiology.

[CR169] Russell J, Jarrold C (1999). Memory for actions in children with autism: self versus other. Cognitive Neuropsychiatry.

[CR170] Schacter DL, Addis DR (2007). The cognitive neuroscience of constructive memory: remembering the past and imagining the future. Philos Trans R Soc B.

[CR171] Schedlbauer AM, Capara MS, Watrous AJ, Ekstrom AD (2014). Multiple interacting brain areas underlie successful spatiotemporal memory retrieval in humans. Scientific Reports.

[CR172] Schlichting ML, Preston AR (2015). Memory integration: neural mechanisms and implications for behavior. Current Opinion in Behavioral Sciences.

[CR173] Semino, S., Ring, M., Bowler, D. M., & Gaigg, S. B. (2017). The influence of task demands, verbal ability, and executive functions on item and source memory in autism spectrum disorder. *Journal of Autism and Developmental Disorders*, 10.1007/s10803-017-3299-6 10.1007/s10803-017-3299-6 PMC5760601 28921058

[CR174] Shimamura AP (2010). Hierarchical relational binding in the medial temporal lobe: the strong get stronger. Hippocampus.

[CR175] Simons JS, Spiers HJ (2003). Prefrontal and medial temporal lobe interactions in long-term memory. Nature Reviews Neuroscience.

[CR176] Simons JS, Garrison JR, Johnson MK (2017). Brain mechanisms of reality monitoring. Trends in Cognitive Sciences.

[CR177] Simons JS, Peers PV, Mazuz YS, Berryhill ME, Olson IR (2010). dissociation between memory accuracy and memory confidence following bilateral parietal lesions. Cerebral Cortex.

[CR178] Skinner EI, Fernandes MA (2007). Neural correlates of recollection and familiarity: A review of neuroimaging and patient data. Neuropsychologia.

[CR179] Skinner EI, Fernandes MA (2010). Effect of study context on item recollection. The Quarterly Journal of Experimental Psychology.

[CR180] Smith BJ, Gardiner JM, Bowler DM (2007). Deficits in free recall persist in Asperger’s Syndrome despite training in the use of list-appropriate learning strategies. Journal of Autism and Developmental Disorders.

[CR181] Solomon M, Frank MJ, Smith AC, Ly S, Carter CS (2011). Transitive inferences in adults with autism spectrum disorders. Cognitive, Affective, & Behavioral Neuroscience.

[CR182] Solomon M, McCauley JB, Iosif AM, Carter CS, Ragland JD (2016). Cognitive control and episodic memory in adolescents with autsm spectrum disorders. Neuropsychologia.

[CR183] Solomon M, Ozonoff SJ, Ursu S, Ravizza S, Cummings N, Ly S, Carter CS (2009). The neural substrates of cognitive control deficits in autism spectrum disorders. Neuropsychologia.

[CR184] Solomon M, Ragland JD, Niemdam TA, Lesh TA, Beck JS, Matter JC, Frank MJ, Carter CS (2015). Atypical learning in autism spectrum disorders: A functional magnetic resonance imaging study of transitive inference. Journal of the American Academy of Child & Adolescent Psychiatry.

[CR185] Souchay C, Wojcik DZ, Williams HL, Crathern S, Clarke P (2013). Recollection in adolescents with Autism Spectrum Disorder. Cortex.

[CR186] South M, Ozonoff S, Suchy Y, Kesner RP, McMahon WM, Lainhart JE (2008). Intact emotion facilitation for nonsocial stimuli in autism: is amygdala impairment in autism specific for social information?. Journal of the International Neuropsychological Society.

[CR187] Southwick JS, Bigler ED, Froehlich A, DuBray MB, Alexander AL, Lange N, Lainhart JE (2011). Memory functioning in children and adolescents with autism. Neuropsychology.

[CR188] Spaniol J, Davidson PS, Kim AS, Han H, Moscovitch M, Grady CL (2009). Event-related fmri studies of episodic encoding and retrieval: Meta-analyses using activation likelihood estimation. Neuropsychologia..

[CR189] Spreng RN, Stevens WD, Chamberlain JP, Gilmore AW, Schacter DL (2010). Default network activity, coupled with the frontoparietal control network, supports goal-directed cognition. Neuroimage.

[CR190] Tanweer T, Rathbone CJ, Souchay C (2010). Autobiographical memory, autonoetic consciousness, and identity in Asperger sydrome. Neuropsychologia.

[CR191] Toichi M, Kamio Y (2002). Long-term memory and levels-of-processing in autism. Neuropsychologia.

[CR192] Toichi M, Kamio Y, Okada T, Sakihama M, Youngstrom EA, Findling RL, Yamamoto K (2002). A lack of self-consciousness in autism. American Journal of Psychiatry.

[CR193] Trontel HG, Duffield TC, Bigler ED, Abildskov TJ, Froehlich A, Prigge MBD, Travers BG, Anderson JS, Zielinski BA, Alexander AL, Lange N, Lainhart JE (2015). Mesial temporal lobe and memory function in autism spectrum disorder: an exploration of volumetric findings. Journal of Clinical and Experimental Neuropsychology.

[CR194] Tulving E (1985). Memory and consciousness. Canadian Psychology.

[CR195] Tulving E, Thomson DM (1973). Encoding specificity and retrieval processes in episodic memory. Psychological Review.

[CR196] Uddin LQ, Supekar K, Lynch CJ, Cheng KM, Odriozola P, Barth ME, Phillips J, Feinstein C, Abrams DA, Menon V (2015). Brain state differentiation and behavioral inflexibility in autism. Cerebral Cortex.

[CR197] Vilberg KL, Rugg MD (2012). The neural correlates of recollection: Transient versus sustained FMRI effects. The Journal of Neuroscience.

[CR198] Wais PE, Rubens MT, Boccanfuso J, Gazzaley A (2010). Neural mechanisms underlying the impact of visual distraction on retrieval of long-term memory. Journal of Neuroscience.

[CR199] Whitehouse AJO, Mayberry MT, Durkin K (2007). Evidence against poor semantic encoding in indivduals with autism. Autism.

[CR200] Wilkinson DA, Best CA, Minshew NJ, Strauss MS (2010). Memory awareness for faces in individuals with autism. Journal of Autism and Developmental Disorders.

[CR201] Williams D (2010). Theory of own mind in autism: Evidence of a specific deficit in self-awareness?. Autism.

[CR202] Williams DL, Goldstein G, Minshew NJ (2006). The profile of memory function in children with autism. Neuropsychology.

[CR203] Williams D, Happé F (2009). Pre-conceptual aspects of self-awareness in autism spectrum disorder: the case of action-monitoring. Journal of Autism and Developmental Disorders.

[CR204] Williams DL, Minshew NJ, Goldstein G, Mazefsky CA (2017). Long-term memory in older children/adolescents and adults with autism spectrum disorder. Autism Research.

[CR205] Wojcik DZ, Moulin CJA, Souchay C (2013). Metamemory in children with autism: Exploring “feeling-of-knowing” in episodic and semantic memory. Neuropsychology.

[CR206] Wojcik DZ, Waterman AH, Lestie C, Moulin CJA, Souchay C (2014). Metacognitive judgments-of-learning in adolescents with autism spectrum disorder. Autism.

[CR207] Yazar Y, Bergström ZM, Simons JS (2014). Continuous theta burst stimulation of angular gyrus reduces subjective recollection. PLoS ONE.

[CR208] Yonelinas AP (2001). Consciousness, control, and confidence: the 3 Cs of recognition memory. Journal of Experimental Psychology: General.

[CR209] Yonelinas AP (2002). The Nature of Recollection and Familiarity: A Review of 30 Years of Research. Journal of Memory and Language.

[CR210] Yonelinas AP, Ritchey M (2015). The slow forgetting of emotional episodic memories: An emotional binding account. Trends in Cognitive Sciences.

[CR211] Zalla T, Daprati E, Sav A-M, Chaste P, Nico D, Leboyer M (2010). Memory for self-performed actions in individuals with Asperger syndrome. PloS One.

[CR212] Zeithamova D, Dominck AL, Preston AR (2012). Hippocampal and ventral medial prefrontal activation during retrieval-mediated learning supports novel inference. Neuron.

